# Isolation, Identification and Prevention of Bacterial Spot Disease on *Grifola frondosa*

**DOI:** 10.3390/jof11110777

**Published:** 2025-10-28

**Authors:** Jun-Tao Ge, Na Rong, Jia-Zhe Li, Yao-Yao Lu, Shi-Yi Tao, Xin-Ru Ye, Jun-Xia Cheng, Jia-Qi Wang, Bo Zhang, Yu Li, Jia-Jun Hu

**Affiliations:** 1 College of Life Science, Zhejiang Normal University, Jinhua 321004, China; gjt0707@hotmail.com (J.-T.G.); 15712665776@163.com (J.-Z.L.); 19548990227@163.com (Y.-Y.L.); 18368883993@163.com (S.-Y.T.); 13588134826@163.com (X.-R.Y.); wangjiaqi@zjnu.edu.cn (J.-Q.W.); 2 Science and Research Center for Edible Fungi of Qingyuan County, Lishui 323800, China; immortalrong@163.com; 3 Jinhua Center for Disease Control and Prevention, Jinhua 321000, China; cheng_Junxia97@163.com; 4 Engineering Research Center of Edible and Medicinal Fungi, Ministry of Education, Jilin Agricultural University, Changchun 130118, China; zhangbofungi@126.com (B.Z.); fungi966@126.com (Y.L.); 5 Industrial Development Institute for Plants, Animals and Fungi Integration of Biyang County, Zhumadian 463700, China

**Keywords:** mushroom diseases, *Priestia aryabhattai*, host range, chemical control, biological control, cell wall disruption

## Abstract

*Grifola frondosa* is a rare fungus valued for its nutritional and medicinal properties; however, its bacterial spot disease has been largely overlooked. Thus, this study systematically investigated, isolated, and identified the pathogen and evaluated control strategies for bacterial spot disease affecting *G. frondosa* cultivation in Qingyuan County, Zhejiang Province. Through integrated morphological, physiological and biochemical analysis, and multi-locus phylogenetic analyses (16S rRNA, *gyrB*), *Priestia aryabhattai* was identified as the causal pathogen. This pathogen exhibited host specificity, infecting only *G. frondosa* and *Pleurotus ostreatus*, inducing primordial growth arrest and causing spots on the stipe of mature fruiting bodies. Control assessments revealed significant antimicrobial efficacy for four chemical agents, benziothiazolinone, copper sulfate, ethylicin and tetramycin, three plant extracts, garlic, leek and onion, and two biocontrol strains, *Chlorophyllum molybdites* and *Aspergillus fumigatus*. Scanning electron microscopy (SEM) demonstrated that these treatments caused ultrastructural damage to the pathogen’s cells, including membrane shrinkage, depression, and perforation. These findings establish key pathogenic characteristics and provide a scientific foundation for integrated disease management, supporting sustainable *G. frondosa* cultivation.

## 1. Introduction

China’s edible fungi industry commenced a period of substantial growth in 1978, when it accounted for merely 5.7% of global production (approximately 58,000 metric tons). By 2013, its output had surged to 31.697 million metric tons, representing over 70% of the world’s total. This share further increased to surpass 80% by 2020 [1,2]. Within China’s agricultural sector, the industry is consistently ranked as the fifth-largest by output value, establishing itself as a key pillar within crop cultivation after grain, oil crops, vegetables, and fruits [1,3]. Since the turn of the 21st century, China has accelerated its global expansion by capitalizing on its advanced industrialized production capabilities. In 2017, the nation accounted for 42% of global factory-based output, thereby cementing its dominant position in the international market [1]. *Grifola frondosa* (Dicks.) Gray, a rare fungus known for its nutritional and medicinal properties, possesses cultivation potential and has been successfully cultivated on an industrial scale [1]. Beyond rich concentrations of high-quality proteins, essential vitamins, and trace elements, *G. frondosa* polysaccharides exhibit pharmacological activities including antitumor and immunomodulatory effects, positioning it as a resource for functional foods and pharmaceuticals [4]. Thus, *G. frondosa* is widely cultivated in China, such as Hebei, Yunnan, Zhejiang, and other provinces, with Qingyuan County in Zhejiang Province maintaining the highest production capacity nationwide [5].

As the cultivation scale of *G. frondosa* has expanded, the incidence of diseases has significantly increased, severely affecting both yield and quality [6,7,8]. For example, in Qingyuan County, Zhejiang Province, the primary production region, pathogenic diseases have emerged as a major constraint to the development of the industry. Bacterial diseases, in particular, are highly detrimental, causing substantial economic losses [9]. For instance, bacterial diseases pose serious threats to the cultivation of *G. frondosa*. In Guangdong Province, China, infection by *Bacillus cereus* causes severe decay of the primordia, characterized by foul odor, extensive whitish mucus, and bubble formation, leading to stunted growth or complete failure of fruiting body development, which significantly reduces yield [7]. Concurrently, bacterial spot disease, primarily caused by *Pseudomonas* spp., results in yellowish to brownish rust spots on the pileus. These spots rapidly expand into sunken brown lesions with a putrid odor, causing desiccation, malformation, and cracking of the fruiting bodies, thereby severely compromising product quality and market value [8]. Likewise, the cultivation of *G. frondosa* in Qingyuan County is seriously threatened by bacterial pathogens. This is compellingly evidenced by a recent first report from Qingyuan County, Zhejiang Province, where *Burkholderia gladioli* was identified as the causal agent of a bacterial blotch disease, with field incidence rates reaching approximately 40% and causing significant quality and yield losses [10]. However, compared to fungal diseases, research on bacterial diseases lags far behind.

Due to its simplicity and rapid efficacy, chemical control remains the dominant approach, with major agents including antibiotics, copper-based compounds, and azole fungicides [11]. These chemicals act through multiple mechanisms: oxidative damage disrupts cellular structures, altered membrane permeability induces leakage of cellular contents, and metabolic inhibition interferes with biosynthetic pathways. For example, benzothiazole derivatives employ a multi-target strategy, concurrently inhibiting key processes including cell wall biosynthesis (e.g., DprE1), cell division (FtsZ), and DNA replication (GyrB/ParE). This synergistic mechanism underlies their potent activity against drug-resistant pathogens like MRSA and VRE [12].

However, chemical control methods exhibit several critical limitations that undermine their effectiveness and sustainability. These include inefficiency in utilization, persistent environmental contamination, development of pathogen resistance, and inherent toxicity, all of which pose significant risks to both ecosystem and human health while conflicting with sustainable agricultural practices. A case study from Lishui City demonstrates these concerns, where edible mushrooms contained dithiocarbamate residues at 0.165 mg/kg (13.8% detection rate), indicating potential chronic health hazards for consumers [13]. Compounding this issue, excessive application of fungicides like carbendazim has rapidly selected for resistant pathogen strains. Most alarmingly, 33.67% of China’s edible mushroom exports fail to meet international residue standards, directly threatening the industry’s long-term viability [13]. These empirical observations collectively demonstrate the fundamental deficiencies of chemical control approaches regarding their operational efficiency, safety profile, and environmental sustainability.

In contrast, biological control offers distinct advantages through its dual functionality in disease suppression and plant growth promotion. This approach employs three primary classes of biocontrol agents: (i) phytochemicals [14] such as allicin, (ii) microbial antagonists [15] including *Streptomyces* Waksman and Henrici species and *Trichoderma* Pers. fungi, and (iii) fungal-derived secondary metabolites like saponins and sesquiterpenes [16]. For instance, the microbial antagonist *Trichoderma harzianum* Rifai not only provides effective disease control through direct mycoparasitism and enzymatic degradation of pathogens but also promotes plant growth, achieving over 100% yield increase in tomato. This efficacy can be amplified through genetic engineering or integration with chemical methods, showcasing the adaptable and synergistic potential of biological control [17]. Collectively, these biological agents exert antimicrobial effects through multiple mechanisms—including membrane disruption and metabolic interference—while stimulating plant development. This integrated approach combines high efficacy with environmental safety and minimal resistance development, establishing a sustainable paradigm for crop protection systems.

This study first isolates, identifies and reports *P. aryabhattai* as the causal pathogen of bacterial spot disease in *G. frondosa* by integrating morphological studies, phylogenetic analysis, physiological and biochemical characteristics, while also elucidating its host specificity. In addition, chemical and biological control measures were evaluated, screening four high-efficiency chemical agents, three botanical extracts, and two biocontrol strains.

## 2. Materials and Methods

### 2.1. Pathogenic Bacteria Isolation

Symptomatic fruiting bodies of *G. frondosa* displaying characteristic bacterial blotch symptoms were collected from Qingyuan County Edible Fungi Research Center. The collection was conducted during a disease survey carried out between October and November of 2023. Samples were immediately placed in sterile containers, transported to the laboratory on ice, and processed within 24 h. The fruiting bodies were initially surface-sterilized with 75% ethanol, followed by three rinses with sterile distilled water, and then transferred to sterile Petri dishes [18]. Diseased tissue samples were aseptically excised from the lesion margins, inoculated onto Nutrient Agar (NA) plates (peptone 10 g; beef extract 3 g; sodium chloride 5g; agar 15g; distilled water 1000 mL; pH 7.1–7.5), and incubated at 28 °C in complete darkness. Following incubation, distinct colonies were purified through successive streak plating on fresh NA medium until axenic cultures were obtained. Then, the purified strains were stored at 4 °C for future use [19].

### 2.2. Morphological Observation

For pathogen identification, the purified strain was used for observation. Gram staining was performed to characterize the isolates, along with microscopic examination of specialized cellular structures, including flagella, capsules, and endospores, using specific reagents [20].

For biocontrol fungi, the identification was referred to the Atlas of Chinese Macrofungal Resources [21]. Dried specimens were rehydrated in 94% ethanol and subsequently mounted in 3% potassium hydroxide (KOH) for examination. Structures such as basidiospores, basidia, and cheilocystidia were observed.

The biocontrol microorganisms were streaked onto Gauze’s Synthetic Medium No. 1 (soluble starch 20 g, KNO_3_ 1 g, NaCl 0.5 g, K_2_HPO_4_·3H_2_O 0.5 g, MgSO_4_·7H_2_O 0.5 g, FeSO_4_·7H_2_O 0.01 g, distilled water 1000 mL, pH 7.4–7.6) and incubated at 28 °C for 7–14 d. During this period, its growth characteristics, including colony morphology and production of diffusible pigments, were observed. Then, the micro-characteristics, such as aerial hyphae, spore filaments, and spores, were examined.

### 2.3. Physiological and Biochemical Analysis

To gain a further understanding of the pathogen’s growth characteristics, the following physiological and biochemical experiments were performed: sugar fermentation, nitrate reduction, gelatin liquefaction, starch hydrolysis, citrate utilization, hydrogen sulfide production, motility (stab inoculation), urease activity, indole production, methyl red test, oxidation-fermentation (O/F) test, and Voges–Proskauer (V-P) test [22].

### 2.4. PCR and Phylogenetic Analysis

Genomic DNA was extracted using the TIANamp Micro DNA kit (Tiangen Biotech (Beijing) Co., Ltd., Beijing, China), and the genome extraction quality was determined by 1% agarose gel electrophoresis.

Pathogenic bacteria were characterized using 16S rRNA (16S-27F and 16S-1492R) and gyrB (gyrB-F and gyrB-R); microorganisms were identified using ITS (ITS1 and ITS4) [23], *BenA* (Bt2a and Bt2b) [23], and *CaM* (CMD5 and CMD6) [23]; and macrofungi were identified using ITS (ITS1 and ITS4), *tef1-ɑ* (EF-983F and EF-1567R) [24], and nLSU (LROR and LR5) [25]. PCR amplifying system (25 µL): 9 µL dd H_2_O, 12.5 µL 2 × Es Taq MasterMix (Dye), 1 µL forward primer, 1 µL reverse primer and 1.5 µL genomic DNA. PCR reaction conditions: 94 °C pre-denaturation for 5 min, followed by 94 °C denaturation for 30 s, 52 °C (nLSU, *tef1-ɑ*), 55 °C (ITS, *benA*, *CaM*), 56 °C (16S), and 58 °C (*gyrB*), annealing for 45 s, 72 °C extension for 1 min, a total of 35 cycles, and finally 72 °C extension for 10 min. The PCR amplification products were confirmed by 1% agarose gel electrophoresis and then sent to Beijing Qingke Biotechnology Co., Ltd. Beijing, China, for sequencing.

The newly obtained sequences were submitted to NCBI GenBank https://www.ncbi.nlm.nih.gov/genbank/. (accessed on 2 September 2025) for BLAST analysis, the same and similar species were selected and downloaded, and the multiple sequence comparison was performed by BioEdit 7.2.5 [26]. The phylogenetic analysis was performed using both maximum likelihood analysis (ML) and Bayesian inference (BI). ML was performed through RAxML 8.0.26 [27] and BI was performed using MrBayes 3.2 [28]. And the sequences used in this study are listed in Table 1, Table 2 and Table 3.

### 2.5. Pathogenicity Tests

The surfaces of both original and differentiated *G. frondosa* fruiting bodies were mechanically wounded using sterile needles. The pathogen suspension was then inoculated at the wound sites and incubated at 25 °C under controlled humidity. Sterile water served as the negative control. Each treatment was performed in triplicate (three independent fungal bags), and fruiting body formation was regularly monitored and documented. The pathogen was then reisolated from the infected fruiting bodies and subjected to morphological characterization and phylogenetic analysis to fulfill Koch’s postulates [29].

### 2.6. Host Range Test

The widely cultivated mushrooms *Pleurotus eryngii* (DC.) Quél., *Hericium erinaceus* (Bull.) Pers., *Pleurotus ostreatus* (Jacq.) P. Kumm., *F. filiformis* (Z.W. Ge, X.B. Liu & Zhu L. Yang) P.M. Wang, Y.C. Dai, E. Horak & Zhu L. Yang, and *Hymenopellis raphanipes* (Berk.) R.H. Petersen were selected for the host range test. Mechanical wounding was applied to the pileus and stipes of test mushrooms at both primordial formation and fruiting body development stages using sterile needles after disinfection. Then, the pathogen suspension was uniformly sprayed onto the inoculation sites until it reached a dripping state. Sterile distilled water served as the negative control.

### 2.7. Effects of Chemical–and Bio–Compounds on Pathogen

Six chemical compounds and six biological pesticides were selected for testing (Table 4). All compounds were serially diluted with sterile distilled water to generate concentration gradients of 125, 250, 500, 1000, and 2000 mg/L [30] for further experiments.

Then, the Oxford cup method [31] was employed to evaluate the inhibitory effect of the agents on pathogens. A volume of 200 μL of each agent, at varying concentrations, was added into an Oxford cup on an NA plate inoculated with the pathogen and incubated at 28 °C for 2–3 d. Each concentration was evaluated in triplicate, with sterile distilled water serving as the negative control. The inhibition zone was then measured using cross-section method. The inhibition rate, virulence regression equation, and median effective concentration value (EC50) [32] were calculated through SPSS Statistics 27.

### 2.8. Effectiveness of Plant Extracts in Controlling Pathogens

Fresh garlic (*Allium sativum* L.), onion (*Allium cepa* L.), leek (*Allium porrum* L.), chrysanthemum (*Chrysanthemum morifolium* Ramat.), dandelion (*Taraxacum mongolicum* Hand.-Mazz.), and peony (*Paeonia lactiflora* Pall.) were selected, and the extractions were referred to the reference [33,34,35,36,37], then stored at 4 °C. Then, the Oxford cup method was employed to evaluate the inhibitory effect of the extractions on pathogens. A volume of 200 μL of each extraction was added into an Oxford cup on an NA plate inoculated with the pathogen, followed by incubation at 28 °C for 2–3 d. The inhibition zone was then measured using the cross-section method. All treatments in the assay were performed in triplicate, with sterile distilled water serving as the negative control.

### 2.9. Research on the Effectiveness of Biological Control Agents

A co-culture assay was employed for primary screening. In this assay, agar plates were first spread with 100 μL of a pathogenic fungal suspension. After drying, a 5 mm plug of a candidate biocontrol microorganism was inoculated at the center of the plate. The plates were incubated at 28 °C for 2–3 d, and isolates that formed a clear inhibition zone were selected as potential antagonists. After, based on the result of the co-culture assay, the fermentation broth was tested for its bacteriostatic activity using the Oxford cup method, with each process repeated three times.

### 2.10. Effects of Different Chemicals, Plant Extracts, and Biocontrol Microbiology Treatments on Pathogen

Bacterial samples were collected from the inhibition zone using a flame-sterilized toothpick and transferred onto carbon-coated adhesive tabs mounted on aluminium stubs. The samples were then sputter-coated with a 5 nm gold–palladium layer for 30 s using an ion sputter coater. Subsequently, the specimens were examined under a Zeiss EVO LS10 scanning electron microscope (SEM, Carl Zeiss AG, Oberkochen, Germany) at an accelerating voltage of 5 kV, and digital images were captured at 10,000× magnification.

## 3. Results

### 3.1. Pathogen Identification

#### 3.1.1. Morphological Characteristics of the Pathogen

**Figure 1:**
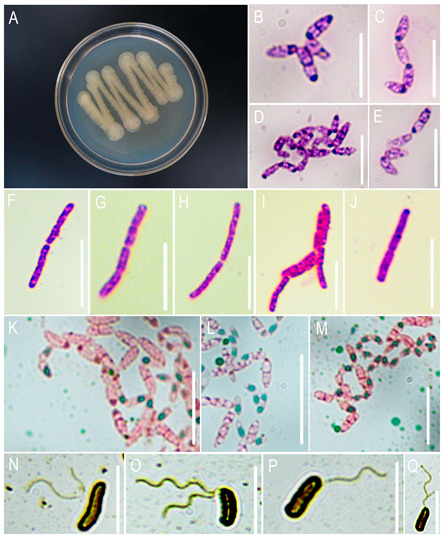
Micro-morphological and special staining identification of the pathogenic bacteria. (**A**) Bacterial lawn; (**B**–**E**) capsules; (**F**–**J**) Gram-staining results; (**K**–**M**) endospores; (**N**–**Q**) flagella. Bars: 5 μm.

Colonies appeared pale yellow with moist surfaces on NA, flat, and margins smooth (Figure 1A), Gram-positive staining (Figure 1F–J), capsules absent (Figure 1B–E), flagella present (Figure 1N–Q), endospores round to oval (Figure 1K–M), typically terminal or subterminal, without significant sporangium swelling. These characteristics match the description of *Priestia aryabhattai.*

#### 3.1.2. Physiological and Biochemical Characteristics of the Pathogen

**Figure 2:**
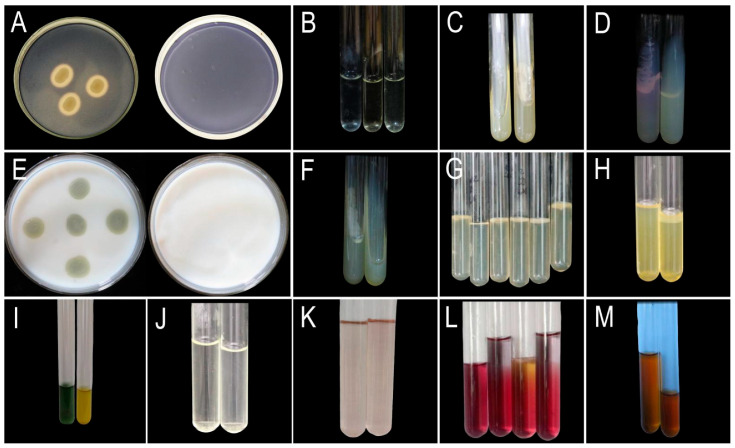
Physiological and biochemical characterization of pathogenic bacteria. (**A**) Starch hydrolysis (left: experimental group; right: negative control). (**B**) Nitrate reduction test (left: negative control; middle: experimental; right: experimental + zinc dust). (**C**) Citrate utilization test (left: negative control; right: experimental). (**D**) Urea hydrolysis (left: negative control; right: experimental). (**E**) Casein hydrolysis (left: experimental; right: negative control). (**F**) Hydrogen sulfide production (left: negative control; right: experimental). (**G**) Motility stab test (tubes 1, 3–6: experimental; tube 2: negative control). (**H**) Gelatin liquefaction (tubes 1–2: experimental; tube 3: negative control). (**I**) Mannitol fermentation (left: negative control; right: experimental). (**J**) Voges–Proskauer test (left: negative control; right: experimental). (**K**) Indole production test (left: negative control; right: experimental). (**L**) Oxidation-Fermentation (O/F) test (Tube 1: Glycerol-free control; Tube 2: Glycerol-supplemented control; Tube 3: Glycerol-free experimental; Tube 4: Glycerol-supplemented experimental). (**M**) Methyl red test (left: control; right: experimental).

The results (Figure 2, Table 5) demonstrated that the pathogen exhibits nitrate reductase activity, gelatinase production, and starch/casein hydrolysis capabilities. Additionally, the pathogen was urease-positive, capable of utilizing mannitol, and exhibited oxidative metabolism. However, it was unable to utilize citrate as the sole carbon source, did not produce hydrogen sulfide, lacked tryptophanase activity, and failed to produce organic acids or neutral end products from glucose metabolism.

#### 3.1.3. Phylogenetic Analysis

**Figure 3:**
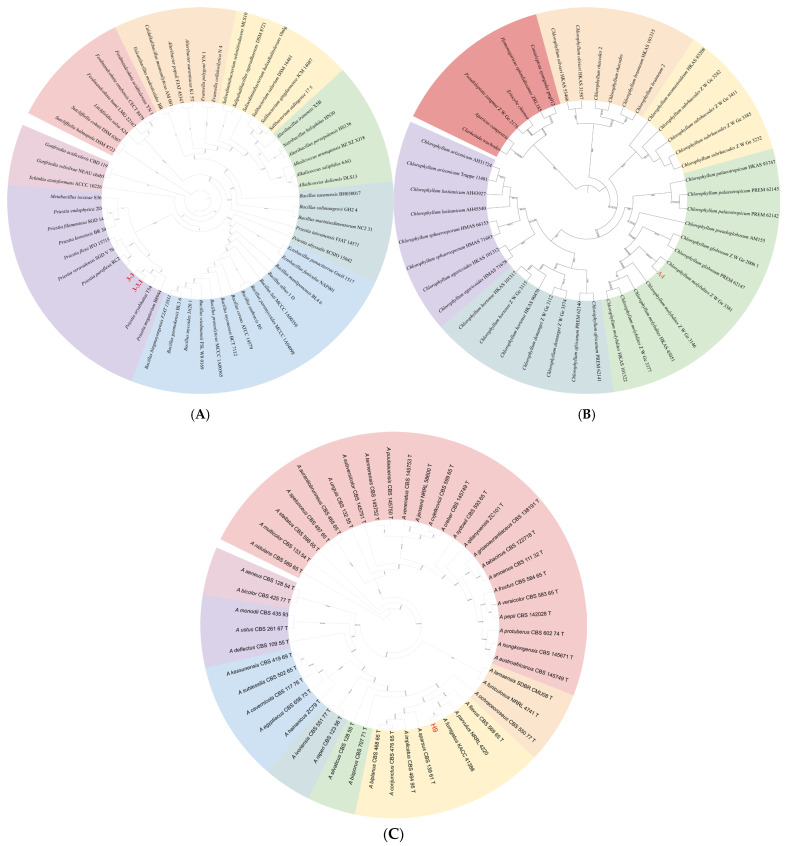
Phylogenetic reconstruction of three biological groups. (**A**) Pathogen inferred from Maximum likelihood (ML) and Bayesian inference (BI) analyses of the combined 16S rRNA and *gyrB* sequence dataset. (**B**) Macrofungi inferred from ML and BI analyses of the combined ITS, nLSU and *tef1-α* dataset. (**C**) Microorganism inferred from ML and BI analyses of the combined ITS, *BenA* and *CaM* dataset. Bootstrap support values (BS) ≥ 80% from ML analysis and Bayesian posterior probabilities (PPs) ≥ 0.90 are shown at the nodes (BS/PP). Color-coded branches represent major taxonomic clades.

The sequenced data were uploaded to NCBI, and the BLAST results revealed a high similarity with *P. aryabhattai*. Phylogenetic analysis, incorporating 16S rRNA and *gyrB* loci, revealed that our sequences clustered within the *P. aryabhattai* clades (Figure 3A) with strong support.

Combined with morphological and biochemical characteristics, along with phylogenetic analysis, the isolate was determined to be *P. aryabhattai*.

### 3.2. Pathogenicity Tests

**Figure 4:**
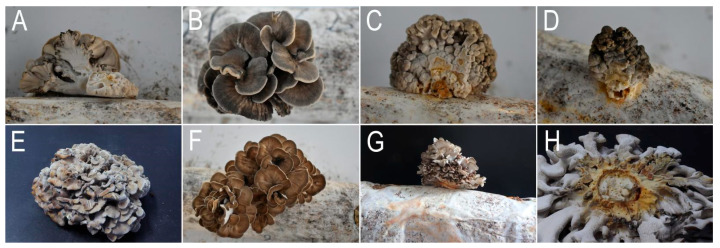
Results of Koch’s postulates verification. (**A**,**B**) Control group; (**C**,**D**) *G. frondosa* infected at primordial stage; (**E**,**F**) stipe of *G. frondosa* infected post-differentiation; (**G**,**H**) pileus of *G. frondosa* infected post-differentiation.

Pathogenicity assays revealed distinct symptom progression based on the timing of infection: infection during primordia formation resulted in complete growth arrest, post-differentiation infections produced basal stipe lesions without involvement of the cap, exhibiting symptom patterns identical to those of field-collected diseased specimens. Control specimens treated with sterile water remained asymptomatic throughout the observation period (Figure 4). The reisolation of the pathogen yielded the same result, thus fulfilling Koch’s postulates.

### 3.3. Host Range Test of the Pathogen

**Figure 5:**
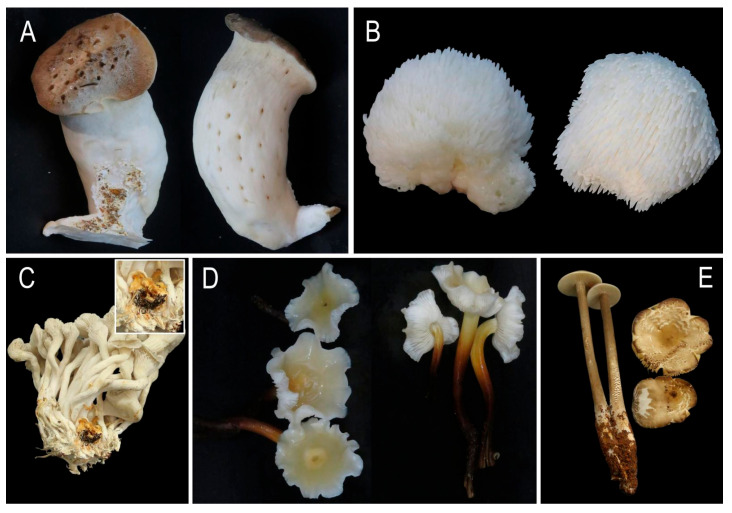
Host range of pathogen in the tested mushrooms. (**A**) *P. eryngii*; (**B**) *H. erinaceus*; (**C**) *P. ostreatus*; (**D**) *F. filiformis*; (**E**) *H. raphanipes*.

Host range assays revealed differential pathogenicity among the tested mushrooms. Only *P. ostreatus* caused complete growth arrest during primordia formation and exhibited spots on the stipe of mature fruiting bodies. No significant symptoms were observed in *P. eryngii*, *H. erinaceus*, *F. filiformis*, and *H. raphanipes* (Figure 5).

### 3.4. Bacteriostatic Effect of Different Chemical and Biological Agents

**Figure 6:**
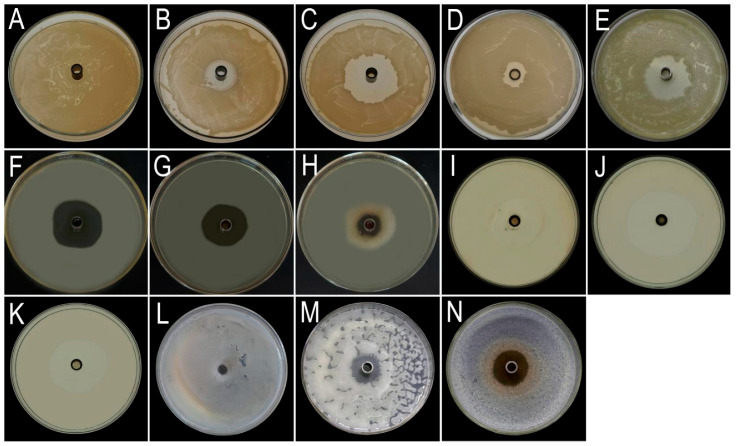
Oxford cup assay screening of antimicrobial agents against target pathogens. (**A**) CK. (**B**–**E**) Chemical agents and biological pesticides: (**B**) copper sulfate, (**C**) benziothiazolinone, (**D**) tetramycin, (**E**) ethylicin. (**F**–**H**) Plant extracts: (**F**) garlic, (**G**) leek, (**H**) onion. (**I**–**K**) Microbial strains: (**I**) H6, (**J**) H9, (**K**) T2. (**L**–**N**) Macrofungal strains: (**L**) CK, (**M**) A3, (**N**) A4.

**Figure 7:**
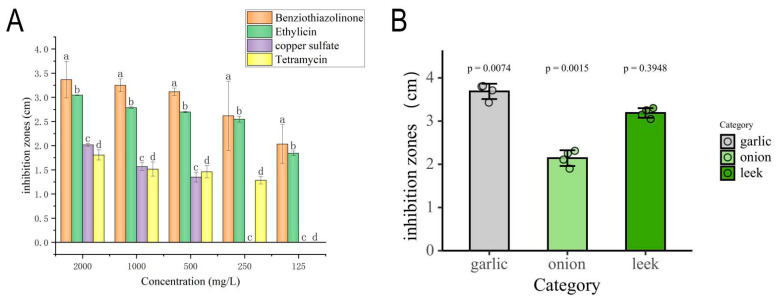
Dose–response profiling of antimicrobial candidates against *P. aryabhattai*. (**A**) Chemical agents and biological pesticides: Inhibition zones of benziothiazolinone (a), ethylicin (b), copper sulfate (c), and tetramycin (d) against target pathogens at concentrations (125–2000 mg/L). Letters indicate significant differences (*p* < 0.05, ANOVA). (**B**) Plant extracts: Comparative efficacy of garlic, onion, and leek extracts with *p*-values from t-tests. Individual circles show data points from biological replicates (n = 3); error bars represent standard deviation.

The results showed that, among the six tested chemical agents, two agents, benziothiazolinone and copper sulfate, showed significant effects (Figure 6B,C and Figure 7A). Benzisothiazolinone demonstrated the most potent antibacterial activity, producing an inhibition zone with a diameter of 25.7 ± 1.7 mm. Copper sulfate was the second most effective agent, forming an inhibition zone measuring 12.3 ± 0.3 mm. In contrast, the remaining chemical agents exhibited no detectable antibacterial effects.

Among the six biological agents tested, only ethylicin and tetramycin inhibited pathogen growth (Figure 6C,D). Ethylicin was the most effective, producing an inhibition zone of 22.5 ± 0.4 mm. Tetramycin demonstrated the second strongest efficacy, with a zone diameter of 9.6 ± 1.5 mm. In contrast, the remaining agents exhibited no significant antibacterial activity.

Toxicity regression models (Table 6) revealed copper sulfate exhibited the narrowest acute toxicity range, while Ethylicin showed the broadest. Pathogen sensitivity was confirmed by EC50 values <5 mg/L for all effective agents, indicating strong antimicrobial activity.

### 3.5. The Effectiveness of Plant Extracts in Controlling Pathogens

Antimicrobial assays demonstrated significant growth inhibition of the pathogen by the following tested extracts (Figure 6F–H and Figure 7B). Garlic extract exhibited the strongest antibacterial activity, producing a mean inhibition zone of 34.3 ± 2.1 mm, followed by leek (32.5 ± 2.5 mm) and onion (21.2 ± 1.8 mm). In contrast, the remaining plant extracts exhibited no significant antibacterial activity.

### 3.6. Biocontrol Microorganism Screening

**Figure 8:**
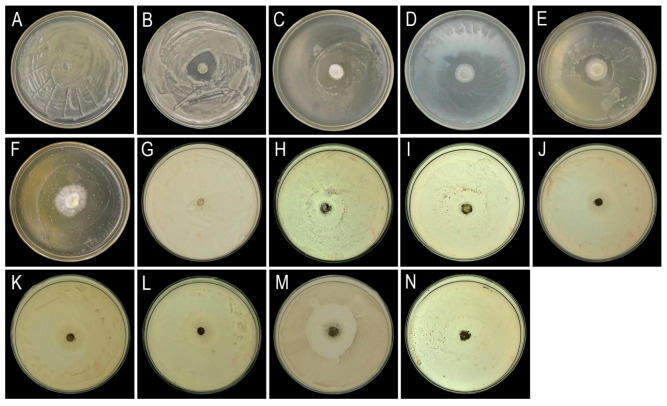
Results of the co-culture screening assay. Macrofungal strains (**A**–**F**): (**A**) CK; (**B**) A1; (**C**) A2; (**D**) A3; (**E**) A4; (**F**) A5. Microorganism strains: (**G**–**N**): (**G**) CK; (**H**) B6; (**I**) B12; (**J**) H6; (**K**) H7; (**L**) H9; (**M**) T2; (**N**) T5.

Primary screening using co-culture methods identified five macrofungal strains (A1–A5) and seven microbial strains (B6, B12, H6, H7, H9, T2, T5) (Figure 8) with inhibitory effects on the pathogen growth. Then, secondary screening via Oxford cup assays revealed two macrofungal strains and three microbial strains exhibiting strong inhibitory effects. Among these, macrofungal strain A4 and microbial strain H9 demonstrated the strongest inhibitory activity, with inhibition zones measuring 33.6 ± 0.5 mm and 45.2 ± 0.9 mm, respectively.

### 3.7. Identification of Biocontrol Microorganisms

#### 3.7.1. Identification of Biocontrol Macrofungi A4

**Figure 9:**
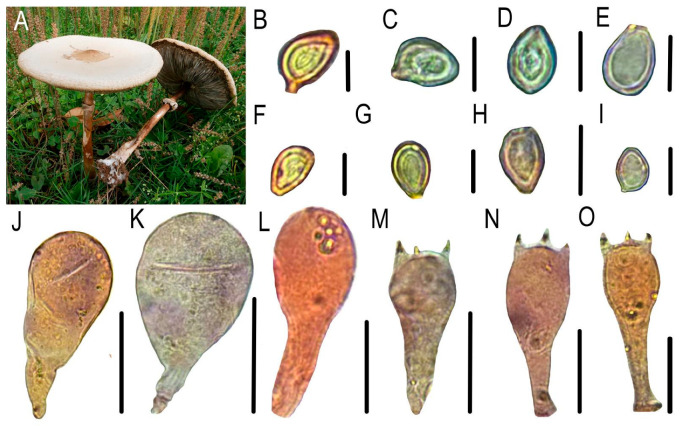
Morphological characteristics of *C. molybdites*. (**A**) Habitat, (**B**–**I**) basidiospores, (**J**–**L**) cheilocystidia, (**M**–**O**) basidia. Bars: 10 μm.

Basidiomata medium to large, pileus convex to planate, adorned with brown scales, lamellae free, closed, greenish, stipe smooth, hollow, cylindrical, enlarged at base, annulus persistent, single-layered, up-part, context white, fleshy, changes to red when injured. Basidiospores ellipsoid, light green, thick-walled, (8.5)9–10(10.5) × 6.5–8 μm. Basidia clavate, 27–36 × (9.5)11–14(16) μm, hyaline, thin-walled, two or four sterigmata (Figure 9). These characteristics match the description of *Chlorophyllum molybdites*.

BLAST analysis revealed that the sequences of A4 exhibited 100% similarity to *C. molybdites*. Furthermore, phylogenetic analysis based on the combined ITS, *tef1-ɑ*, and nLSU fragments clustered A4 within the *C. molybdites* clade, with strong support (Figure 3B). Combined with the results of morphological studies and phylogenetic analysis, it was identified as *C. molybdites*.

#### 3.7.2. Identification of Antagonistic Microbial Strain H9

**Figure 10:**
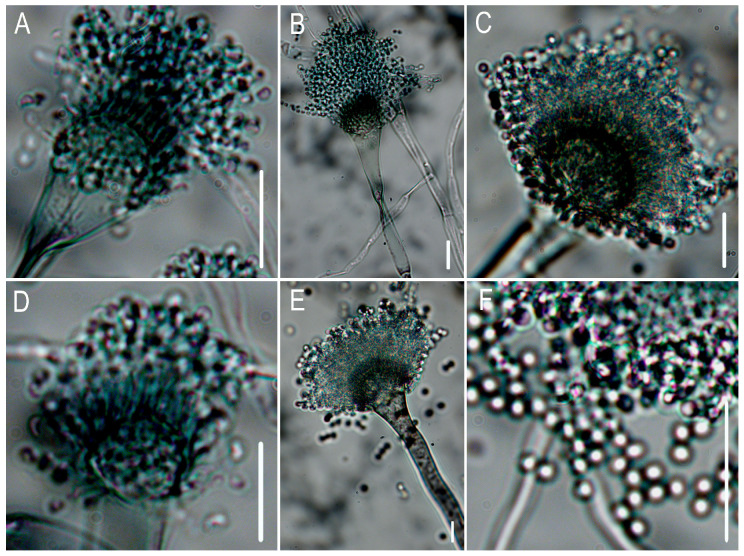
Morphological characterization of *Aspergillus fumigatus*. (**A**–**E**) Conidiophores (**F**) conidia. Bars: 10 μm.

Following 7 d of incubation on Czapek–Dox medium, microscopic examination identified foot cells. Conidial heads were predominantly columnar (44.30–72.02 μm × 36.92–42.06 μm) with characteristic grayish-green pigmentation. Conidiophores appeared smooth-walled, hyaline to pale brown, and cylindrical (4.58–10.05 μm; 150–250 μm). Vesicles were consistently flask-shaped (10.87–32 μm diameter). Conidia arising from phialides on the vesicles were subhyaline, subglobose to broadly ellipsoidal (1.84–2.05 μm diameter), with finely echinulate surfaces (Figure 10). It matches the descriptions of *Aspergillus fumigatus*.

The BLAST results revealed a high similarity with *A. fumigatus*. Phylogenetic analysis, incorporating ITS, *benA* and *CaM* loci, revealed that our sequences clustered within the *A. fumigatus* clade (Figure 3C) with strong support. Combined with the results of morphological identification and phylogenetic analysis, it was identified as *A. fumgatus*.

### 3.8. The Influence of Different Agents on Pathogens

**Figure 11:**
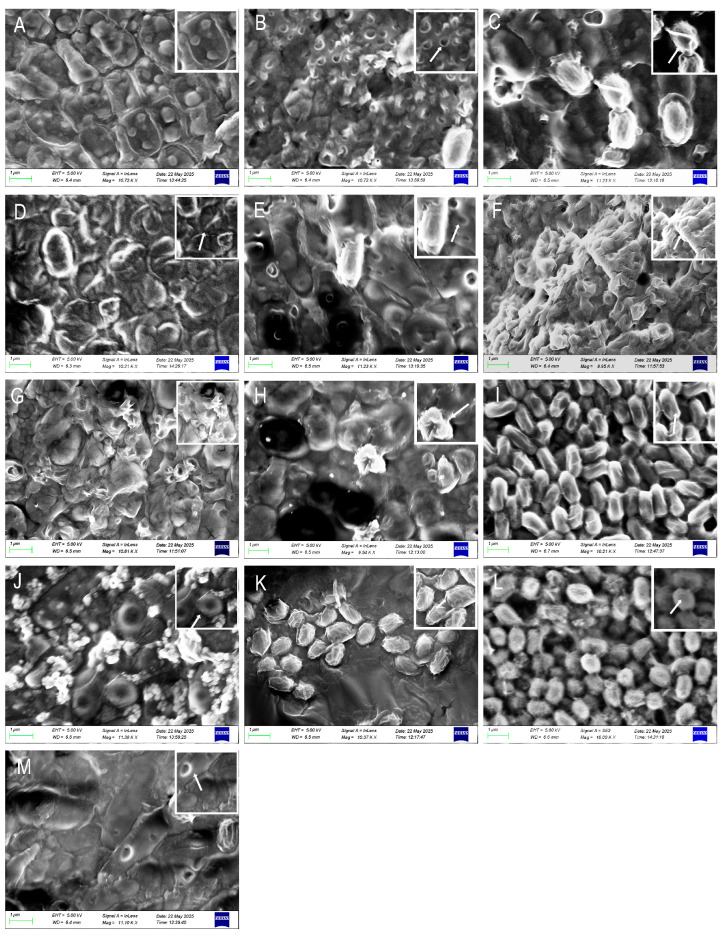
Ultrastructural alterations in microbial cells induced by diverse antimicrobial treatments (**A**) CK; (**B**) copper sulfate; (**C**) benziothiazolinone; (**D**) tetramycin; (**E**) ethylicin; (**F**) garlic; (**G**) leek; (**H**) onion; (**I**) A3; (**J**) A4; (**K**) H6; (**L**) H9; (**M**) T2.

SEM analysis revealed distinct, agent-specific morphological alterations in pathogen cells following exposure to antimicrobial treatments. Cells treated with benziothiazolinone or tetramycin exhibited pronounced surface wrinkling and perforations, a phenotype that was also produced by metabolites from strains H9 and H6 (Figure 11C,D,I–M). Conversely, copper sulfate and ethylicin induced significant membrane depression and extensive perforation, a damage pattern similarly elicited by metabolites from strains A4 and A3 (Figure 11B,E,F–H). These ultrastructural changes, including membrane indentation and pore formation, were dose-dependent. In contrast, untreated control cells maintained a typical, intact cellular architecture with no signs of disruption (Figure 11A).

## 4. Discussion

Bacterial spot disease poses a significant challenge in the cultivation of *G. frondosa*, severely compromising its yield and quality. This study investigated bacterial spot disease of *G. frondosa* from Qingyuan County, Zhejiang Province, China, isolating and identifying the pathogen, determining its host range, and evaluating chemical and biological control measures. Through Koch’s postulates, this study identified *P. aryabhattai* as the causative pathogen of *G. frondosa* bacterial spot disease. The pathogen displays a narrow host range, primarily infecting *G. frondosa* and *P. ostreatus* under our experimental conditions, where it induces growth arrest at the primordia stage and exhibits spots on the stipe of mature fruiting bodies. Notably, it exhibited no detectable pathogenicity toward other tested edible fungi, including *H. erinaceus*, *F. filiformis* and *H. raphanipes*. This study further explored chemical and biological control strategies, identifying one microbial strain and one macrofungal species with significant antagonistic activity against *P. aryabhattai*. Notably, *A. fumigatus* (H9) and *C. molybdites* (A4) demonstrated the most potent inhibition. Among the three tested plant extracts, garlic extract showed the strongest antimicrobial activity. Additionally, benziothiazolinone and ethylicin were identified as effective chemical control agents for disease management. SEM revealed that the aforementioned treatments resulted in significant structural damage to the cell surface of *P. aryabhattai*, including shriveling, depressions, and perforations.

### 4.1. The Influence of Cell Wall on Pathogen Host Specificity

Host range determination revealed that *P. aryabhattai* is highly pathogenic to *P. ostreatus* (Figure 5C) and *G. frondosa* (Figure 4), while it remains non-pathogenic or shows negligible symptoms toward other tested edible mushroom species (Figure 5). This host specificity may stem from distinct pathogen-host cell wall interactions. *Grifola frondosa*, *P. ostreatus*, *P. eryngii*, *H. erinaceus*, *F. filiformis* and *H. raphanipes* exhibit substantial variations in their cell wall polysaccharide composition and structure, which likely determine differential susceptibility to *P. aryabhattai* infection.

Both *G. frondosa* and *P. ostreatus* possess similar β-(1,3)-glucan main chains with β-(1,6)-branching structures [4]. These soluble polysaccharides may serve as specific recognition sites or enzymatic targets for pathogenic bacteria, potentially explaining their shared susceptibility to *P. aryabhattai* infection. The mycelium of *G. frondosa* contains abundant low-molecular-weight (13–140 kDa) β-(1,3)-glucans with high-density β-(1,6)-branches, which significantly enhance pathogenic adhesion and enzymatic degradation efficiency [38]. *P. ostreatus* shares the same glucan backbone structure; its medium molecular weight polysaccharides and protective hydrophobic protein layers provide partial physical resistance [39], resulting in comparatively milder infection symptoms.

Notably, although *P. ostreatus* and *P. eryngii* are taxonomically related, their cell wall polysaccharides demonstrate fundamental structural differences. While *P. ostreatus* retains a characteristic β-(1,3)-glucan core structure, *P. eryngii* uniquely substitutes this with an α-(1,3)-galactan backbone featuring 3-O-methylation modifications. This structural reorganization confers enhanced spatial stability through steric hindrance effects, providing a mechanistic basis for the observed resistance to *P. aryabhattai* colonization [40,41].

Furthermore, structural analyses reveal distinct cell wall compositions among resistant species: *H. erinaceus* synthesizes β-glucans with a β-(1→6)-linked backbone (85.7% abundance), incorporating β-(1→3)-linked branch points (14.3%). These β-(1→3) linkages interconnect multiple β-(1→6) chains, forming a branch-on-branch topological network. Enzymatic digestion confirmed that oligosaccharides containing β-(1→3) bonds (e.g., dp5f: G1→3G1→6G1→3G1→6G) resist degradation due to steric hindrance, conferring structural rigidity [42], while *F. filiformis* synthesizes α-configured glucans featuring a predominantly α-(1→4)-glycosidic backbone, interspersed with α-(1→6)-linked branching points (average: one branch per 12 glycosyl units). These α-(1→6) bonds serve as topological nodes for interchain cross-linking, organizing multiple α-(1→4) chains into a sparsely branched network. Enzymatic digestion assays confirmed that oligosaccharide domains containing α-(1→6) branches—particularly those terminating in galactose or fucose residues—resist degradation via steric hindrance, thereby imparting conformational stability to the macromolecular structure [43]. Meanwhile, the cell wall of *H. raphanipes* consists primarily of a β-glucan backbone (72.69%), modified with arabinose (1.09%) and other functional groups. This structure forms a physical barrier via β-glycosidic linkages, where arabinose residues may facilitate cross-linking stabilization [44]. Experimental evidence validated that the polysaccharide inhibits specific binding interactions with signalling pathway proteins [45]—such as suppression of β-catenin nuclear translocation—suggesting a resistance mechanism against enzymatic degradation by pathogens.

Therefore, we speculate that *P. aryabhattai* may achieve infection by selectively degrading the cell wall polysaccharides of *G. frondosa* and *P. ostreatus* through glucanase targeting β-1,3 bonds, while the different cell walls of *P. eryngii*, *H. erinaceus*, and *F. filiformis* resist pathogen invasion through physical barriers and biochemical resistance.

### 4.2. Structural Disruption of the Cell Wall: A Unifying Antibacterial Mechanism for Diverse Control Agents

This study systematically evaluated various disease control strategies, including chemical bactericides, biological control agents, plant-derived antimicrobials, and antagonistic microorganisms. Screening assays identified benzothiazolinone and ethylicin as highly effective compounds, demonstrating potent antibacterial activity against *P. aryabhattai*. Additionally, of SEM results revealed significant structural damage to the surface of the treated pathogen cells, including shrivelling, depressions, and perforations (Figure 11). The cell wall, which acts as a key barrier in maintaining cell shape and resisting osmotic pressure, is essential for cell integrity. Any loss of this integrity leads to cell rupture, leakage of cellular contents, and ultimately, the inhibition or death of pathogens.

Mechanistic investigations revealed that both agents exert their antimicrobial effects primarily by compromising the bacterial cell wall: benziothiazolinone inhibits peptidoglycan synthesis, while ethylicin disrupts membrane-associated lipoproteins. Among these compounds, ethylicin exerts its potent oxidative effects primarily through active sulfur derivatives produced during decomposition (e.g., ethyl thiosulfinate). These derivatives target sulfur-containing functional groups in microbial cell walls and membranes, disrupting the synthesis and cross-linking of cell wall components or directly degrading their integrity, leading to structural deformation and eventual collapse. Furthermore, upon penetrating the cell membrane, ethylicin disrupts the functionality of membrane-associated lipids and proteins, increasing permeability and inducing protoplasmic leakage. Collectively, these mechanisms inhibit pathogenic growth [46].

The core antibacterial mechanism of benziothiazolinone (BIT) lies in its role as a multi-target metabolic inhibitor, as evidenced by its diverse effects on cellular structures and functions. BIT rapidly penetrates the bacterial cell membrane and enters the cytoplasm, where it irreversibly binds to the active sites of various key enzymes—particularly thiol-containing dehydrogenases. This is corroborated by recent findings in fungi, where BIT significantly inhibits NADH production and disrupts mitochondrial integrity, leading to fragmentation [47]. This binding leads to the fatal disruption of cellular energy metabolism and respiratory processes, as directly evidenced by a sharp decrease in intracellular ATP levels [47], ultimately causing physiological dysfunction due to energy deprivation. Such internal biochemical disturbances trigger secondary effects, including the failure of systems responsible for maintaining membrane integrity. Direct evidence from studies on the bacterium *Xanthomonas oryzae* pv. *oryzae* confirms that BIT compromises the structural organization and barrier function of the cell membrane, resulting in cell wrinkling, distortion, and the leakage of intracellular contents, as clearly observed via scanning and transmission electron microscopy [48]. Furthermore, these primary effects cascade into the inhibition of vital processes such as DNA replication, biofilm formation, and motility [48], underscoring its multi-target nature.

Notably, conventional copper-based compounds exhibited limited efficacy in this screening assay, suggesting the presence of copper ion resistance in the pathogen. This finding highlights the importance of novel mechanisms of action demonstrated by thiabendazole and allicin, which specifically target cell wall degradation. As a result, these compounds offer a promising strategy to overcome resistance to conventional chemical treatments [12].

Plant-derived extracts from garlic, leek, and onion exhibit significant antibacterial activity. SEM observations revealed distinct morphological changes in pathogenic bacteria, including surface indentations and perforation-like structures (Figure 11F–H). These antimicrobial effects are likely mediated by the lipophilic and chemically reactive organosulfur compounds present in these extracts, particularly allicin [49] and its structural analogs (e.g., methyl allyl trisulfide and propyl sulfide), which display potent bioactive properties.

These compounds integrate into the phospholipid bilayer of the cell membrane through lipophilic groups, reducing membrane fluidity and causing localized collapse. As the concentration increases, they further disrupt the spatial conformation of membrane protein complexes (such as pore proteins and transport proteins), forming irreversible pores, leading to leakage of intracellular ions, such as K^+^ and Ca^2+^, and the outflow of macromolecular substances [50]. This results in osmotic imbalance and membrane potential depolarization. After penetrating the cell membrane, active sulfur groups (such as thiosulfate esters) covalently bind to essential enzymes containing sulfhydryl groups (-SH), forming disulfide bonds [49]. This irreversible protein denaturation inhibits DNA replication, repair, and antioxidant functions, ultimately leading to the disruption of cellular structure.

During the screening of biocontrol microorganisms, highly antagonistic strains of *C. molybdites* and *A. fumigatus* were isolated. SEM analysis demonstrated that treatment with these microbial antagonists caused significant morphological changes in *P. aryabhattai*, including localized invaginations and irregular surface corrugations of the cellular envelope (Figure 11J,L). Among these, *Aspergillus fumigatus* mediates antibacterial effects via secretion of the anionic defensin AfusinC. This molecule specifically binds lipid II, a key peptidoglycan precursor, inhibiting cell wall biosynthesis and inducing osmotic lysis through loss of structural integrity. Concentration-dependent bactericidal activity against Gram-positive bacteria (e.g., Micrococcus luteus and Staphylococcus aureus) was observed. At 4 × MIC (16 μg/mL), 48 h exposure reduced bacterial loads by ≥3 log_10_ CFU/mL, with MBC values 1−4 × MIC, indicating direct lytic action. AfusinC’s function requires its conserved CSαβ motif (disulfide-stabilized α-helices/β-sheets), where cationic residues (e.g., Lys-32) electrostatically target membrane components. The defensin’s anionic character (net charge: −1.5) enables Gram-positive selectivity, while Gram-negative bacteria remain resistant due to outer membrane barriers. This cell wall synthesis-targeting mechanism contrasts with nonspecific membrane disruption by cationic antimicrobial peptides [51]. In contrast, *C. molybdites* exerts its antibacterial effect through the secretion of toxins. Saponin toxins interact with phospholipids through hydrophobic interactions, leading to the localized collapse of the cell wall’s network structure. Additionally, sesquiterpene toxins disrupt the lipid bilayer, forming transmembrane pores that result in membrane potential collapse and osmotic imbalance [16,52].

*Grifola frondosa*, a valuable edible and medicinal macrofungus, holds significant economic potential owing to its distinctive nutritional composition and pharmacological properties. This study systematically identifies the bacterial pathogen responsible for spot disease in *G. frondosa* cultivation. Additionally, we have screened multiple highly effective biocontrol agents, laying the groundwork for sustainable disease management in *G. frondosa* cultivation. Moving forward, it is essential to conduct field trials to evaluate whether these biocontrol agents remain effective in controlling *G. frondosa* under varying environmental conditions. Furthermore, an in-depth investigation of the antibacterial mechanisms in different control materials is required, alongside the use of response surface methodology to optimize the fermentation of selected antagonistic bacteria. The development of microbial pesticides, the exploration of synergistic chemical and biological control strategies, and the establishment of an integrated pest management system that is both environmentally friendly and economically viable will promote the sustainable growth of *G. frondosa* industry.

## Figures and Tables

**Table 1:** Species and sequences used in phylogenetic analyses of pathogenic bacteria. Sequences produced in this study are in bold.

Species	Strain	GenBank Accession No.	
		16S rRNA	*gyrB*
*Alkalicoccus daliensis*	DLS13	GU583651	
*A. saliphilus*	6AG	AJ493660	
*A. urumqiensis*	BZ SZ XJ18	KM066107	
*A. iranensis*	X5B	HQ433452	
*A. persepolensis*	HS136	FM244839	
*Alteribacter aurantiacus*	K1-5	AJ605773	
*A. populi*	FJAT 45347	KY612313	
*Bacillus albus*	1(D)	MW240406.1	MK816869.1
*B. anthracis*	B5	MH384783.1	MH427049.1
*B. bingmayongensis*	FJAT-13831	JN885201.1	JN874726.1
*B. cereus*	ATCC 14579	MH806388	
*B. gaemokensis*	BL3-6	FJ416489	
*B. luti*	MCCC 1A00359	KJ812415	
*B. manliponensis*	BL4-6	FJ416490	
*B. marinisedimentorum*	NC2-31	KX548355	
*B. mycoides*	JA20-1	PP101188.1	PP107863.1
*B. paramycoides*	MCCC 1A04098	KJ812444	
*B. proteolyticus*	MCCC 1A00365	KJ812418	
*B. solimangrovi*	GH2-4	KC616733	
*B. taeanensis*	BH030017	AY603978	
*B. toyonensis*	BCT 7112	AJ310100	
*B. wiedmannii*	FSL W8 0169	KU198626	
*Caldalkalibacillus mannanilyticus*	AM 001	AB043864	
*Ectobacillus funiculus*	NAF001	AB049195	
*E. panaciterrae*	Gsoil 1517	AB245380	
*Evansella cellulosilytica*	N 4	AB043852	
*E. polygoni*	YN-1	AB292819	
*Fredinandcohnia aciditolerans*	YN-1	MG589508	
*F. humi*	LMG 22167	AJ627210	
*F. onubensis*	CECT 8479	LN650668	
*Gottfriedia acidiceleris*	CBD 119	DQ374637	
*G. solisilvae*	NEAU cbsb5	KJ733017	
*Litchfieldia salsa*	A24	HQ433466	
*Metabacillus iocasae*	S36	KY462210	
*Natribacillus halophilus*	HN30	AB449109	
*Priestia abyssalis*	SCSIO 15042	JX232168	
*P. aryabhattai*	T54	PV174529.1	PV554262.1
***P. aryabhattai***	**3-3**	**PX225877**	**PX242661**
***P. aryabhattai***	**3-3.1**	**PX247483**	**PX264259**
*P. endophytica*	2D	AF295302	
*P. filamentosa*	SGD 14	KF265351	
*P. flexa*	IFO 15715	AB021185	
*P. koreensis*	BR 30	FJ889614	
*P. megaterium*	B8944	OR145150.1	PP700230.1
*P. paraflexa*	RC2	FN999943	
*P. pseudoflexus*	RC1	FN999944	
*P. taiwanensis*	FJAT 14571	KF040588	
*P. veravalensis*	SGD V 76	KF413434	
*Salibacterium aidingense*	17-5	DQ504377	
*S. qingdaonense*	JCM 14087	AB571874	
*S. salarium*	DSM 16461	AB571873	
*Salipaludibacillus agaradhaerens*	DSM 8721	X76445	
*Salisediminibacterium haloalkalitolerans*	10nlg	HG934298	
*S. selenitireducens*	MLS10	AF064704	
*Schinkia azotoformans*	ACCC 10226	AB363732	FJ009514.1
*Vulcanibacillus modesticaldus*	BR	AM050346	
**Outgroups**			
*Sutcliffiella halmapala*	DSM 8723	X76447	
*S. cohnii*	DSM 6307	X76437	

**Table 2:** Species and sequences used in phylogenetic analyses of macrofungi. Sequences produced in this study are in bold.

Species	Strain	GenBank Accession No.		
		ITS	nLSU	*tef1-α*
*Chlorophyllum africanum*	PREM 62140	MG741961	MG742041	MG742098
*C. africanum*	PREM 62141	MG741963	MG742042	MG742099
*C. agaricoides*	HKAS 101312	MG742003	MG742020	MG742078
*C. agaricoides*	HMAS 71678	MG742004	MG742021	MG742079
*C. arizonicum*	AH31724	KR233490	KR233490	
*C. arizonicum*	Trappe 11481 (AZ80)	HQ020416	HQ020419	
*C. brunneum*	HKAS 101315	MG742013	MG742022	MG742080
*C. brunneum*		AY083206	AF482886	HM488886
*C. demangei*	Z.W.Ge3112	MG741965	MG742027	MG742084
*C. demangei*	Z.W.Ge 3574	MG741964	MG742025	MG742083
*C. globosum*	Z.W.Ge 2006-1	MG741995	MG742023	
*C. globosum*	PREM 62147	MG742002	MG742024	MG742081
*C. hortense*	HKAS 101317	MG741967	MG742026	MG742082
*C. hortense*	Z.W.Ge 3115	MG741968	MG742028	MG742085
*C. hortense*	HKAS 90470	MG741971	MG742029	MG742086
*C. lusitanicum*	AH45540	KR233482	KR233482	
*C. lusitanicum*	AH43927	KR233483	KR233492	
*C. molybdites*	HKAS 45051	MG741985	MG742030	MG742087
*C. molybdites*	Z.W.Ge 3381	MG741993	MG742034	MG742091
*C. molybdites*	Z.W.Ge 3146	MG741987	MG742031	MG742088
*C. molybdites*	HKAS 101322	MG741988	MG742032	MG742089
*C. molybdites*	Z.W.Ge 3377	MG741992	MG742033	MG742090
***C. molybdites***	**A4**	**PX225872**	**PX226006**	**PX242658**
*C. neomastoideum*	HKAS 83208	MG741976	MG742035	MG742092
*C. olivieri*	HKAS 31587	MG742016	MG742036	MG742093
*C. olivieri*	HKAS 53466	MG742017	MG742037	MG742094
*C. palaeotropicum*	PREM 62142	MG741978	MG742038	MG742095
*C. palaeotropicum*	PREM 62145	MG741982	MG742039	MG742096
*C. palaeotropicum*	HKAS 93747	MG741983	MG742040	MG742097
*C. pseudoglobosum*	AM155	KP642506	KR080484	
*C. rhacodes*		AF482849	AY176345	HM488885
*C. rhacodes*		U85312	U85277	KC884736
*C. sphaerosporum*	HMAS 66153	MG742011	MG742043	MG742100
*C. sphaerosporum*	HMAS 71683	MG742012	MG742044	MG742101
*C. subrhacodes*	Z.W.Ge 3411	MG741975	MG742045	MG742102
*C. subrhacodes*	Z.W.Ge 3232	MG741973	MG742046	MG742103
*C. subrhacodes*	Z.W.Ge 3385	MG741972	MG742048	MG742105
*C. subrhacodes*	Z.W.Ge 3242	MG741974	MG742047	MG742104
**Outgroups**				
*Agaricus campestris*		KM657927	KR006607	KR006636
*Clarkeinda trachodes*		HM488751	KY418837	
*Coniolepiota spongodes*	Png 012	HM488756	HM488774	HM488883
*Eriocybe chionea*		HM488753	HM488772	
*Hymenagaricus splendidissimus*	ZRL 185	HM488760	HM488769	KT951657
*Pseudolepiota zangmui*	Z.W.Ge 2175	KY768928	MG742049	MG742106

**Table 3:** Species and sequences used in phylogenetic analyses of microorganism. Sequences produced in this study are in bold.

Species	Strain	GenBank Accession No.		
		ITS	*BenA*	*CaM*
*Aspergillus aeneus*	CBS 128.54 T	EF652474	EF652298	EF652386
*A. amoenus*	CBS 111.32 T	EF652480	JN853046	JN854035
*A. aurantiobrunneus*	CBS 465.65 T	EF652465	EF652289	EF652377
*A. bicolor*	CBS 425.77 T	EF652511	EF652335	EF652423
*A. biplanus*	CBS 468.65 T	EF661210	EF661116	EF661130
*A. bisporus*	CBS 707.71 T	EF661208	EF661121	EF661139
*A. cavernicola*	CBS 117.76 T	EF652508	EF652332	EF652420
*A. conjunctus*	CBS 476.65 T	EF661179	EF661111	EF661133
*A. cvjetkovicii*	CBS 599.65 T	EF652440	EF652264	EF652352
*A. deflectus*	CBS 109.55 T	EF652437	EF652261	EF652349
*A. egyptiacus*	CBS 656.73 T	EF652504	EF652328	EF652416
*A. fructus*	CBS 584.65 T	EF652449	EF652273	EF652361
*A. flavus*	CBS 569.65 T	AF027863	EF661485	EF661508
*A. fumigatus*	KACC 41388	JN943563	AY685147	AY689331
***A. fumigatus***	**H9**	**PX225874**	**PX242659**	**PX242660**
*A. griseoaurantiaceus*	CBS 138191 T	KJ775553	KJ775086	KJ775357
*A. hainanicus*	ZC79 T	OM414846	OM475626	OM475630
*A. hongkongensis*	CBS 145671 T	AB987907	LC000552	MN969320
*A. implicatus*	CBS 484.95 T	FJ491656	FJ491667	FJ491650
*A. ivoriensis*	CBS 551.77 T	EF652441	EF652265	EF652353
*A. jensenii*	NRRL 58600 T	JQ301892	JN854007	JN854046
*A. kassunensis*	CBS 419.69 T	EF652461	EF652285	EF652373
*A. lamaensis*	SDBR-CMU08 T	MW388211	MW219783	MW219781
*A. monodii*	CBS 435.93 T	FJ531150	FJ531171	FJ531142
*A. multicolor*	CBS 133.54 T	EF652477	EF652301	EF652389
*A. nidulans*	CBS 589.65 T	EF652427	EF652251	EF652339
*A. ochraceoroseus*	CBS 550.77 T	EF661224	EF661113	EF661137
*A. parvulus*	NRRL 4741 T	EF661273	EF661112	EF661175
*A. pepii*	CBS 142028 T	KU613368	KU613371	KU613365
*A. protuberus*	CBS 602.74 T	EF652460	EF652284	EF652372
*A. puulaauensis*	CBS 145750 T	JQ301893	JN853979	JN854034
*A. qilianyuensis*	ZC101 T	OM414847	OM475627	OM475631
*A. raperi*	CBS 123.56 T	EF652454	EF652278	EF652366
*A. silvaticus*	CBS 128.55 T	EF652448	EF652272	EF652360
*A. sparsus*	CBS 139.61 T	EF661181	EF661125	EF661173
*A. spelunceus*	CBS 497.65 T	EF652490	EF652314	EF652402
*A. stellatus*	CBS 598.65 T	EF652426	EF652250	EF652338
*A. subtessilis*	CBS 502.65 T	EF652485	EF652309	EF652397
*A. subversicolor*	CBS 145751 T	JQ301894	JN853970	JN854010
*A. sydowii*	CBS 593.65 T	EF652450	EF652274	EF652362
*A. tabacinus*	CBS 122718 T	EF652478	EF652302	EF652390
*A. tennerensis*	CBS 145752 T	JQ301895	JN853976	JN854017
*A. unguis*	CBS 132.55 T	EF652443	EF652267	EF652355
*A. ustus*	CBS 261.67 T	EF652455	EF652279	EF652367
*A. venenatus*	CBS 145753 T	JQ301896	JN854003	JN854014
*A. versicolor*	CBS 583.65 T	EF652442	EF652266	EF652354
*A. austroafricanus*	CBS 145749 T	JQ301891	JN853963	JN854025
*A. creber*	CBS 145749 T	JQ301889	JN853980	JN854043
**Outgroup**				
*A. nidulans*	CBS 589.65 T	EF652427	EF652251	EF652339

**Table 4:** Reagents and commercial sources used in this study.

Reagent	Manufacturer
20% thiophanate-copper SC/chemical compounds	Zhejiang Longwan Chemical Co., Ltd. (Wenzhou, China)
50% prochloraz-manganese chloride complex/chemical compounds	Guangzhou Zhonglong Chemical Co., Ltd. (Guangzhou, China)
30% zinc thiazole SC/chemical compounds	Zhejiang Xinnong Chemical Co., Ltd. (Hangzhou, China)
copper sulfate/chemical compounds	Taixing Smelting Plant Co., Ltd. (Taizhou, China)
40% miconazole nitrate/chemical compounds	Zhongxin Chemical Co., Ltd. (Wuhan, China)
3% benziothiazolinone ME/chemical compounds	Adama Huifeng Co., Ltd. (Yancheng, China)
2% kasugamycin AS/biological pesticides	Jiangmen Plant Protection Co., Ltd. (Jiangmen, China)
20% streptomycin/biological pesticides	Jiangsu Yiheng Pharmaceutical Co., Ltd. (Zhenjiang, China)
15% tetramycin/biological pesticides	Liaoning Weike Bio-engineering Co., Ltd. (Chaoyang, China)
20% eugenol/biological pesticides	Maanshan Anbao Agricultural Technology Co., Ltd. (Maanshan, China)
12% zhongshengmycin WP/biological pesticides	Fujian Kaili Bio-products Co., Ltd. (Zhangzhou, China)
80% ethylicin EC/biological pesticides	Henan Kebang Chemical Co., Ltd. (Nanyang, China)

**Table 5:** Physiological and biochemical identification results.

Test	Result	Test	Result	Test	Result
Nitrate reduction	+/−	Indole production	−	Hydrogen sulfide	−
Citrate utilization	−	Methyl red	−	Urease activity	+
Gelatin liquefaction	+	Mannitol fermentation	+	Oxidase/Fermentation test	+
Starch hydrolysis	+	Voges–Proskauer test	−		

Note: (+) indicates positive; (+/−) indicates weakly positive; (−) indicates negative.

**Table 6:** Virulence regression curves and EC50 values of four effective reagents.

Reagent	Toxicity Regression Models	EC50 (mg/L)	95%Confidence Interval	Reagent	Toxicity Regression Models	EC50 (mg/L)	95%Confidence Interval
Benziothiazolinone	y = 2.1424 × −4.5657	2.190	2.020–2.214	Ethylicin	y = 1.6151 × −3.3638	2.083	0.702−2.376
Copper sulfate	y = 3.9968 × −11.3475	2.839	2.484–3.213	Tetramycin	y = 2.6285 × 6.7956	2.585	1.269−3.207

## Data Availability

The gene accession numbers referenced in this study are publicly available in the online databases: NCBI GenBank https://www.ncbi.nlm.nih.gov/genbank/. (accessed on 2 September 2025). The reference numbers of the sequences produced in this study are PX225872, PX226006, PX242658, PX225874, PX242659, PX242660, PX225877, PX242661, PX247483 and PX264259.
